# Rural women are more likely to use long acting contraceptive in Tigray region, Northern Ethiopia: a comparative community-based cross sectional study

**DOI:** 10.1186/s12905-015-0229-7

**Published:** 2015-09-04

**Authors:** Mussie Alemayehu, Aster Kalayu, Alem Desta, Hailay Gebremichael, Tesfalem Hagos, Henock Yebyo

**Affiliations:** Department of Public Health, Mekelle University, College of Health Sciences, Mekelle, Ethiopia; JSI/L10K, Tigray region branch, Tigray, Ethiopia; School of Medicine, College of Health Sciences, Mekelle University, Mekelle, Ethiopia

## Abstract

**Background:**

In the latest report of Ethiopian Demographic and Health Survey (EDHS) 2011, the maternal mortality ratio (MMR) was estimated at 676/100,000 live births, with total fertility rate at 4.8 and contraceptive prevalence rate at 29 %. Knowledge and utilization of long acting contraceptive in the Tigray region are low. This study aims at comparing and identifying factors related to the utilization of long acting contraceptive in urban versus rural settings of Ethiopia.

**Methods:**

A comparative community-based cross-sectional study, comprised of quantitative and qualitative methods, was conducted among 1035 married women in Wukro (urban area) and Kilteawlaelo district (rural area) in March, 2013. Stratified sampling technique was employed to approach the study participants. Data were analyzed using SPSS version 20. Multiple logistic regression analysis was used to identify the respective effect of independent predictors on utilization of long acting contraceptive.

**Results:**

The proportion of long acting contraceptive use among the respondents was 19.9 % in the town of Wukro and 37.8 % in the district of Kilteawlaelo. Implanon was the most common type of contraceptive used in both districts, urban (75 %) and rural (94 %). The odds of using the long acting contraceptive method were three times higher among married women in the rural areas as compared with the urban women [AOR = 3. 30; 95 %, CI:2.17, 5.04]. No or limited support from male partners was an obstacle to using long acting contraceptive method [AOR = 0. 24, 95 of CI: 0.13, 0.44]. Moreover, married women whose partner did not permit them to use long acting contraceptive [AOR = 0. 47, 95 % of CI: 0.24, 0.92] and women who attended primary education [AOR = 0.24, 95 %, CI: 0.13, 0.44] were significantly associated with long acting contraceptive use.

**Conclusion:**

Overall, the proportion of long acting contraceptive use has found to be low. Rural women were more likely to use long acting contraceptives as compared to urban women. Moreover, educational status and the partner’s permission to use contraception could influence the utilization of long acting contraceptives.

## Background

On a global scale, maternal mortality is unacceptably high. Every day about 800 women die due to pregnancy and child-birth related complications, almost all of which (99 %) occur in developing countries [1]. Ethiopia is a developing country with a substantial maternal mortality ratio (MMR) of 676/100,000 live births [2]. However, such large numbers of maternal deaths could be averted by using family planning, particularly; long acting methods are more advantageous because of it needs less visit to health care facilities, highly effective and cheap. Moreover, addressing the unmet need of family planning could have averted 12,800 maternal deaths and more than 1.1 million deaths of children [3].

Ethiopia is one of the Sub-Saharan African countries that showed progress in promoting access to family planning services over the past 15 years. Contraceptive use among married women increased from 6 % in 2000 to 42 % in 2014 [4–6]. However, this success is far below the national target to reach a contraceptive prevalence rate of 66 % by 2015 [7]. In 2011, 25 % of married Ethiopian women had an unmet need of family planning, although the country targeted to lower this unmet need to 10 % by 2015 [7, 8]. And this will require a concerted effort to increase the country’s contraceptive prevalence rate (CPR) and shift to the method mix with greater emphasis on long acting contraceptive. The prevalence of long acting contraceptive methods (LACMs) use in Tigray region is as low as 5.6 % for Implanon and no users for an intrauterine contraceptive device (IUCD). The overall prevalence of any contraceptive use in Tigray is 21.2 % [9].

A number of factors contribute to the low utilization of LACMs. These include: a negative attitude towards the practicing of LACMs, fear of side effects, partner objection, medical problems, the unavailability of methods, lack of knowledge, desire to have more children, age, and preference of short acting contraceptive methods [8].

Some studies have researched long acting contraceptives in the region [10–12], however, there are still limited studies on identifying and comparing the proportion and factors affecting long acting contraceptive between urban and rural settings. Rural women have twice as many children as urban women and are less likely to use modern methods of contraception (38 and 56 percent, respectively) than their counterparts [4]. However; the Ministry of Health of Ethiopia (MoHE) currently allows insertion of Implanon by health extension workers (HEW). HEWs are accessible in every sub district of the country, in rural and urban settings. Most (84 %) of the Ethiopian population resides in rural settings. This study compares the utilization of LACMs and associated factors in urban (Wukro Town) and rural (Kilteawlaelo district) setting.

## Methods

A comparative community-based cross-sectional study, comprised of quantitative and qualitative methods of data collection, was conducted in Wukro town and Kilteawlaelo district in March, 2013. Wukro Town has a total population of 35,032, with women of reproductive age accounting for 8,232. Regarding the health service coverage: the town has one public hospital, two health centers, one private clinic and five drug outlets. In contrast, the rural district (Kilteawlaelo) has a total population of 119,772. Women of reproductive age account for 28,146. Moreover, the health service coverage within the rural area: the district has five public health centers, 17 health posts and one private clinic [13]. All women of reproductive age in both the rural and urban areas were considered as source population. In the study, all women within the reproductive age group were included except those who were not voluntary and those critically ill during the data collection.

The sample size was calculated using an Epi-info version 3.5.3. The sample size was determined based on a double population proportion principle on the assumptions of a proportion of LACM use in Mekelle town (12.3 %) [10], a confidence level of 95 %, and a 5 % degree of precision, 80 % power of a test, ratio of exposed to unexposed 1:1 and assuming an odds ratio of 2. We added a 10 % of the calculated sample size to compensate the expected non-response. The total sample was computed to be 1044 married women.

A stratified two stage cluster sampling was used to select the study participants, urban versus rural as the main strata. The sub-districts (Kebeles) were considered as clusters within a stratum. There were a total of three Kebeles in Wukro town and 19 Kebeles in Kilteawlaelo district. We selected two Kebeles from the town at complete random considering the number of eligible women. Likewise, we selected eight Kebeles from the rural side using simple random sampling. We used the households in each of the selected Kebeles as a sample population. Using systematic random sampling, households were selected and women who fulfilled the eligibility criteria were consented for an interview. Whenever two or more eligible women were found in a household, we included only one as the information regarding the LACM could be similar among them.

Furthermore, four sessions of focus group discussion (FGDs) (two FGDs each in married women and men) were carried out to augment the quantitative findings. Each FGD consisted of 8 participants. Purposive sampling technique was used to select the participants by considering sex and residence (urban and rural) of the participants.

The quantitative questionnaire was prepared by referencing previously published literature [2, 10, 11]; but was contextualized into the local setting. It contains information on socio-demographic and economic characteristics, reproductive history, and knowledge and attitude towards utilization of LACMs. It was initially prepared in English and then translated back into the local language (Tigrigna). To ensure consistency of the contents of each question, the questionnaire was back-translated into English by a different person. To check the plausibility of the tool, estimate time for the interview and ensure understandability of the questions, pretest was done on 5 % of married women outside the study areas. The interview was performed face-to-face. Six experienced diploma nurses were used as data collectors and two bachelor nurses were assigned as supervisors for the quantitative portion of the study. Training was given to the data collectors and supervisors for two consecutive days on the purpose of the study, methodology, the contents of the questionnaire, and, particularly, on issues related to the confidentiality of the responses and the rights of respondents.

Focus group discussions were done using a semi-structured topic guide to understand the phenomena of couple’s contraceptive practices within the society. The discussion was tape recorded by facilitators, including the principal investigator, and notes were taken throughout the process.

### Measurement

Women’s attitudes about the use of LACMs were measured by a total of five questions. Questions regarding attitude were grouped into two categories: “strongly agrees/agree” were labeled as “agree,” and “strongly disagree/disagree/neutral” as “disagree”. The mean score was computed to measure the attitudes of the participants towards utilization of long acting contraceptive. Accordingly, respondents who scored above the mean were considered as positive attitude and those below the mean were considered a negative attitude. The prevalence and factors associated LACM with it was used to compare between the urban and rural women of reproductive age group.

### Data analysis

Data were entered, cleaned and analyzed using SPSS 20 for Windows (SPSS Inc. Version 20, Chicago, Illinois). A descriptive analysis was computed for each variable at the initial. Bivariate logistic regression analyses were done for the explanatory variables with the dependent variable to select contender variables for multiple logistic regression models. Those variables found significant at a p-value of less than 0.2 were analyzed for their relative effect using the multiple logistic regression. The effect sizes of the sample were described using adjusted odds ratio (AOR) and that of the population using 95 % CI. A p-value less than 0.05 was set as cut off for the statistical significance. Multi colinarity test was done to see the interaction among the independent variables. Data from the FGDs were translated and transcribed into English and categorized accordingly to the main thematic areas. The findings of the qualitative part were triangulated along with the findings of the quantitative result. All the analysis was done based on comparison of the urban versus rural women.

### Ethical clearance

The ethical review committee of Mekelle University, College of Health Sciences approved the study protocol as well as the verbal consent form for the participants. Informed verbal consent was obtained from study participants after the purposes of the study were explained to them. The right of the respondents to withdraw from the interview was assured. Data was anonymously encoded.

## Result

### Socio-demographic Characteristics

A total of 1035 respondents were included in the study with a response rate of 90.5 %. Fifty percent of the respondents were from Wukro town. The mean age of the respondents was 30.7 years (SD ± 6. 2) in Wukro town and 33 years (SD ± 8) in Kilteawlaelo district. Orthodox Christianity was the predominant religion in both study areas, 98 % in Wukro town versus 99 % in Kilteawlaelo district. Sixty percent of participants in Kilteawlaelo and 20 % in Wukro town were unable to read and write. The average walk time to a health facility was 30 minutes in 95 % of respondents in Wukro town and 62 % in Kilteawlaelo district (Table 1).

**Table 1:** Socio-demographic characteristics in Wukro town and Kilteawlaelo district, 2013/14

Variables	Urban (n = 518)	Rural (n = 517)
	n (%)	n (%)
Age of women		
15-24	86 (16.8)	83 (17)
25-29	155 (30)	117 (23)
30-34	121 (23)	70(14)
35-39	106 (21)	111 (22)
40 or more	50 (9.6)	136 (26)
Religion		
Orthodox	506 (98)	513 (99)
Others^a^	12 (2)	4 (1)
Employment		
Farming	4 (0.8)	422 (82)
Business	80 (15.4)	5 (1)
Daily labor	81 (15.6)	2 (0.4)
Government employment	58 (11)	2 (0.4)
Housewife	295 (57)	86 (16.6)
Husband’s Occupation		
Farming	13 (2.5)	455 (88)
Business	100 (19.3)	10 (2)
Daily labor	95 (18.3)	23 (4.5)
Government employment	222 (43)	16 (3)
Others^b^	88 (17.5)	13 (2.5)
Educational status		
No Education	88 (17)	312 (60)
Read and write (No formal education)	83 (16)	77 (15)
Primary	133 (26)	104 (20)
Secondary or higher	214 (41)	24 (5)
Husband’s Education		
No Education	25 (4.8)	172 (33)
Read and write (No formal education)	70 (14)	136 (26)
Primary	130 (25)	173 (34)
Secondary or higher	293 (56.6)	36 (7)
Walking distance to health facility		
Less than 30 minutes	493 (95)	195 (37.7)
30 minutes-1 hour	25 (5)	188 (36.4)
>1 hour	0	134 (25.9)

^a^Muslim, Catholics and Protestants

^b^Unemployed, pensioned, and live on others’ support

### Obstetric history of the married women

The mean age at first marriage was similar in both study populations: 17.4 years (SD ± 3.3) in Kilteawlaelo district and 18.8 years (SD ± 3.7) in Wukro town. A 30 % of the respondents in Wukro town and 50 % in Kilteawlaelo district had been with their spouse for 16 years or more. The average number of pregnancies of each woman in Wukro Town and Kilteawlaelo district were 3 and 4.7, respectively. Twenty percent of the respondents had a history of abortion in both study areas. When the respondents were asked about their future intention to have children, 50 % of them reported that they need additional children within 4 years in Wukro and 6 years in Kilteawlaelo district (Table 2).

**Table 2:** Obstetric history of married women in Wukro town and Kilteawlaelo district, 2013/14

Variables	Urban	Rural
	n (%)	n (%)
Age at marriage	(n = 518)	(n = 517)
<18	185 (35.7)	241 (46.6)
≥18	333 (64.3)	276 (53.4)
Mean	18.75 ± 3.7	17.4 (±3.3)
Number of pregnancies	(n = 518)	(n = 517)
≤2	220 (42.5)	107 (20.7)
3-4	205 (39.6)	147 (28.4)
≥5	93 (18)	263 (50.8)
Mean	3 (±1.62)	4.7 (±2.46)
Number of abortions	(n = 114)	(n = 115)
1	89 (78)	91 (790
2 or more	25 (22)	24 (21)
Current number of children	(n = 512)	(n = 510)
≤2	255 (49.8)	122 (23.9)
3-4	200 (39)	155 (30.4)
≥5	57 (11)	233 (45.7)
Mean	2.67 (±1.46)	4.2 (±2.14)
Desired number of children	(n = 518)	(n = 517)
≤2	32 (6.2)	6 (1.2)
3-4	302 (58.3)	87 (16.8)
≥5	184 (35.5)	424 (81.7)
Mean	4.22 (±1.1)	5.8 (1.38)
Number of years stay in marriage	(n = 518)	(n = 517)
1-5	102 (19.7)	72 (14.6)
6-10	156 (30)	128 (24.8)
11-15	105 (20.3)	59 (11.4)
16 or beyond	155 (30)	258 (50)

### General Awareness of the respondents about family planning

Ninety percent of the respondents had knowledge of short acting contraceptives (Pills or Injectables). IUCD (Loop) was predominantly known in the urban community versus the rural, 79.3 % vs. 46.6 %; while Implanon was commonly known in the rural (34.2 % vs. 53.4 %). Vasectomy was least known (<1 %) in both study settings. The major sources of information about family planning were hospitals and health centers in Wukro town, in contrast to health posts in the Kilteawlaelo district (Table 3).

**Table 3:** Knowledge about LACM in Wukro town and Kilteawlaelo district, 2013/14

Variables	Urban (n = 518)	Rural (n = 517)
	n (%)	n (%)
Knowledge on type of contraceptive method		
Pills	485(93.6)	458(88.6)
Injectable	487(94)	510(98.6)
Loop (IUCD)	177(34.2)	240(46.4)
Implant	409(79)	273(52.8)
Tubal ligation	26(5)	16(3.1)
Vasectomy	3(0.6)	4(0.8)
Condom	109(21)	17(3.3)
Knowledge on source of FP providing institutions		
Hospital	459(88.6)	185(35.8)
Health center	499(96.3)	378(73.1)
Health post	29(5.6)	408(78.9)
Private clinic	11(2.1)	9(1.8)
Pharmacy	35(6.8)	9(1.8)

### Attitude of respondents towards long acting contraceptives

Eighty six (83 %) respondents in Wukro town and 134 (69 %) in Kilteawlaelo district agreed that long acting contraceptive is exclusively important for married women. Almost all respondents in both study settings agreed that long acting contraceptive is effective in preventing pregnancy. Ninety percent of the respondents in Wukro town and 70 % in Kilteawlaelo district disagreed that they have difficulty of accessing long acting contraceptive. Nearly half of the respondents in both the study settings believed that using a long acting contraceptive has its own risks or side effects (Table 4). The results of FGD also supported this. An FGD participant said that *“……. the health extension workers are now teaching us about the benefits of family planning including long acting ones. But I see some women suffering from heavy bleeding after inserting Implanon and this bothers me not to use the method …….”* [32 year’s old, female, Rural]. Another participant of the FGD also strengthens this idea *“… I think it is not good to use long acting contraceptives as they may cause infertility. One should decide to use it once she has more children. ….”* [28 year’s old, female, Urban].

**Table 4:** Attitude of married women towards use of LACM in Wukro town and Kilteawlaelo district, 2013/14

	Wukro Town (n = 103)			Kilteawlaelo (n = 195)		
Variables	Agree n (%)	Neutral n (%)	Disagree n (%)	Agree n (%)	Neutral n (%)	Disagree n (%)
LACM is important for married women	86 (83)	7 (6.7)	10 (10.3)	134 (68.7)	10 (5.1)	51 (26.2)
LACM effectively prevents pregnancy	86 (83)	7 (6.7)	10 (10.3)	192 (98.5)	1 (0.5)	2 (1)
It is difficult to get LACMs	5 (4.8)	4 (4)	94 (91.2)	47 (24.1)	20 (10.3)	128 (65.6)
There is risk of using LACMs	22 (21.4)	20 (19.4)	61 (59.2)	106 (54.4)	30 (15.4)	59 (30.3)
Intend to use LACM in the future	88 (85.4)	5 (4.9)	10 (9.7)	162 (83.1)	13 (6.7)	20 (10.3)

The composite measure of attitude questions indicated that 50 % in Wukro town and 40 % in Kilteawlaelo district had negative attitude towards utilization of long acting contraceptive.

### Practice of long acting contraceptive

The proportion of long acting contraceptive use was 103(19.9 %) in Wukro town and 195 (37.8 %) in Kiteawelaelo district. The results of FGD substantiated this evidence. An FGD participant said “… *using family planning is good for the health of our wives and our children. It is very difficult to take care of many children, to send them to school and to get medical care if they get sick. Now I encourage my wife to use it…”* [42 years, male, Urban]. Implanon was the most frequent type of contraceptive method employed: used by 77 (75 %) in Wukro town and 184 (94 %) in the Kilteawelao district . The result of FGD was in line with this. One of the FGD participants said *“---I don’t agree that the long acting contraceptives, such as Implanon, have severe side effects*. Rather, I believe that we don’t need repeat visits to health care facilities like we do when using ….”*. As such, I personally used it for 2 years….”* [35 years, female, Rural]. IUCD was practiced by 25 % in Wukro and 6 % in Kilteawelo district. The role of partners in their wives use of long acting methods was also assessed. Only about 70 % of women in Wukro town and 65.7 % in Kilteawlaelo district reported that their partners used to permit them to use LACMs. Similarly, nearly 80 % of women in Wukro town and 70 % of Kilteawlaelo district discussed LACMs with their partners at least once. Information regarding to their future intention shows that 257 (62 %) of the respondents in Wukro town and 160 (49.7 %) in Kilteawlaelo district had intended to use LACMs. Out of the current users, the majority were ages 25–39 years in both areas. The study results indicated that as the numbers of experiencing abortions increases, the users of LACM decreases in both areas. The use of LACM follows different trends, however, in relation to the woman’s number of alive children. In Wukro town, the majority of LACM users were women who had few children (two or less) unlike to women with five or more children in Kilteawlaelo. Almost all (94 %) of LACM users in Wukro town were women who reside within a 30 minute walk to a health facility (Table 5).

**Table 5:** Description of long acting and its users by basic demographic among married women in Wukro town and Kilteawlaelo district, 2013/14

Variables	Urban (n = 518)		Rural (n = 517)	
	Use of LACMs		Use of LACMs	
	Yes	No	Yes	No
	n (%)	n (%)	n (%)	n (%)
Occupation				
Farming	2 (2)	2 (0.5)	170(87.5)	250 (78)
Business	17 (17)	63 (15)	5(2.5)	0.00
Daily labor	10 (10)	71 (17)	291)	0.00
Government employment	13 (13)	45 (11)	4 (2)	0.00
Others	61 (59)	234 (56)	14 (7)	72 (22)
Number of abortion				
1	18 (90)	71 (75.5)	40 (83)	51 (76)
2 or more	2 (10)	23 (24.5)	8 (17)	16 (24)
Desired number of children				
≤2	6 (6)	21 (5)	1 (0.5)	1 (0.3)
3-4	63 (61)	242 (58.5)	30 (15.4)	57 (18)
≥5	34 (33)	151 (36.5)	164 (84)	264 (82)
Walking distance from home to health facility				
<30 min	97 (94)	396 (95)	85 (44)	110 (34)
30 min-1 hour	6 (6)	19 (5)	66 (34)	122 (38)
>1 hour	0	0	44 (23)	90 (28)
Type of LACM used				
Implanon	77 (75 %)	0	184 (94 %)	0
I UCD	26 (25 %)	0	11 (6 %)	0

Preference of short acting contraceptive was the main reason mentioned for not using a LACM in both study areas. The results of FGD also support this finding. An FGD participant said that “… *Some religious leaders teach against using any form of contraception. They tell us it is sinful to prevent pregnancy [of baby] who could be a king or a priest. So, there is a concern with some women for not using family planning, but most mothers and couples are now aware of its benefits including myself and my husband…”* [Age 34, female, Rural]. Moreover, finding from the qualitative portion of the study revealed that frequently mentioned reasons for not using LACMs were prolonged bleeding after using Implanon, fear of infertility and delayed service for removal of the method.

### Factors affecting utilization of LACMs

Place of residence (urban versus rural) was found to be a significant predictor of LACMs’ use. The odds of using LACM in the rural area were three times more in married women than their counterparts (AOR = 3. 30, 95 % of CI: 2.17, 5.04).

Partner involvement in the decision to use a contraceptive also contributes to enhancing the utilization of LACMs. As such, the odds of using a LACM among mothers whose partner did not permit them to use a contraceptive was 76 % less than those who got support from their husbands (AOR = 0. 24, 95 % of CI: 0.13,0.44) (Table 6).

**Table 6:** Predictors of LACM use in Wukro town and Kilteawlaelo district, 2013/14

Variables	LACM use		COR (95) %]	AOR (95 %)
	Yes	No		
	n (%)	n (%)		
Residence				
Urban	103 (34.6)	415 (56.3)	1	1
Rural	195 (65.4)	322 (43.7)	2.44 (1.85,3.23)**	3.30 (2.17,5.04)**
Age of mother				
15-24	36 (12.1)	133 (18)	1	1
25-29	66 (22.1)	206 (28)	1.84 (0.75,1.88)	1.02 (0.60,1.76)
30-34	59 (19.8)	132 (17.9)	1.65 (1.02,2.67)*	1.57 (0.80,3.09)
35-39	68 (22.8)	149 (20.2)	1.67 (1.06,2.89)*	1.14 (0.56,2.30)
≥40	69 (23.2)	117 (15.9)	2.18 (1.36,3.50)*	1.40 (0.64,3.04)
Maternal Education				
No education	142 (47.7)	258 (35)	1	1
Read and write only	108 (36.2)	289 (39.2)	0.68 (0.50,0.92)*	0.98 (0.68,1.48)
Primary education	33 (11.1)	136 (18.5)	0.44 (0.29,0.68)*	0.47 (0.24,0.92)*
Secondary and above	15 (5)	54 (7.3)	0.51 (0.28,0.93)*	0.47 (0.20,1.10)
Number of children				
≤2	80 (26.8)	316 (42.9)	1	1
3-4	101 (33.9)	251 (34.1)	1.59 (1.14,2.23)*	2.26 (0.47,10.78)
≥5	117 (39.3)	170 (23)	2.72 (1.94,3.82)**	2.55 (0.42,15.40)
Attitude towards LACM use				
Negative	111 (37.2)	330 (44.8)	1	1
Positive	187 (62.8)	406 (55.2)	1.37 (1.04,1.81)*	1.20 (0.87,1.64)
Partner permission				
Yes	264 (88.6)	441 (59.8)	1	1
No	34 (11.4)	296 (40.2)	0.19 (0.13,0.28)**	0.24 (0.13,0.44)**

*P <0.05 = Significant ** P < 0.01 = highly significant

## Discussion

Implanon was the most frequently used long acting contraceptive in both districts. The proportion of contraceptive users were higher in the Kilteawlaelo district than Wukro town. Place of residence and husbands’ support to use contraceptives were associated with use of LACMs.

Knowledge of family planning is a prerequisite to obtaining access to and using a suitable contraceptive method timely and effectively. IUCD is predominantly known in the urban area, whereas Implanon is more known in the rural. However, the finding of this study was inconsistent with the finding of a study done in Nigeria. The Nigerian study demonstrated that a decreased awareness of contraceptives in rural areas than in urban [14].

The proportion of LACM use in this study ranges from 19.9 % in the town to 37.7 % in the rural districts. This was consistent with the findings of a study performed in multiple areas of Sub Saharan Africa [15], as well as a study done in Debremarkos town, Ethiopia [16]. However, it was higher as compared with the national report of EDHS, 2011 and a study done in the city of Mekelle (12.3 %) [2, 10]. The increase in utilization may be attributable to the current increase in access; i.e., health extension workers are now able to inset Implanon [17]. This also implies that the government of Ethiopia should strengthen the new initiative of scaling up LACM provision by health extension workers.

In this study, the odd of using LACM was higher in the rural area than the urban. Such good utilization of Implanon in the rural areas has resulted from the efforts of health extension workers who are currently licensed to insert Implanon [17]. Moreover, different non-governmental health organizations, including Marie Stopes and Integrated Family Health Program (IFHP) have strategies for distributing and insertion of LACMs in the rural area through outreach activity. This implies that some amendment of family planning strategies could have a significant effect on utilization of LACMs. However, this finding is inconsistent with a finding of Nigeria, in which the proportion of women who use LACM was higher in urban area than rural area [14].

A significant number of women in both districts had negative attitude towards the utilization of long acting contraceptives. Moreover, this study revealed that attitudes were not statically significant with long acting contraceptives. This is also in line with a study completed in the city of Mekelle [10]. However, a study done in Nigeria revealed that the positive attitude of women toward contraceptives was an important factor for promoting use of LACMs [14]. This may be due to the differences in the attributes used to measure attitude in our study and the study of Nigeria.

This study revealed that as the number of abortions increases the LACM users decrease in both study areas. The numbers of women who have undergone at least one abortion were four times in Wukro town as compared with Kilteawelo district. This possibly indicates that women in the rural were more likely to use long acting contraceptive. Moreover, inserting an IUCD or Implanon immediately after abortion significantly reduces the risk of subsequent abortion [18].

The number of women in primary education was almost two times higher in Kilteawelo district as compared with their urban counterparts. Moreover, in the latest report of EDHS 2011, current contraceptive use increases with a woman’s education. Twenty-two percent of women with no education report current use of any method of contraception, compared with 68 percent of women with more than secondary education [2]. This is also in line with our findings, which state that mothers with primary education were 53 % times more likely to use LACM than those with no education.

Two third of married women in both districts had obtained permission from their husband to use long acting contraceptive. This indicates that male involvement in family planning could be a factor in increasing the number of long acting users. Moreover, a study done in Debremarkos, Ethiopia revealed that mothers who had discussions with their husband were more likely to use family planning than their counterparts [16]. Our finding also in line with this, in which the odd of using LACM was 76 % higher among those who had support from their husband than those who did not got support.

Our study has several limitations. The questionnaires and focus groups did not collect information from healthcare providers or obtain husbands’ perspectives. In addition, due to the nature of the sensitivity of some issues (like income and abortion), the information collected might not reflect the entire truth. However, the study team made efforts to remind participants of the privacy of their information and gained as much knowledge as possible.

## Conclusion

The proportion of LACM use is low. Rural women are more likely to use long acting contraceptive than those from the urban area. The husbands’ support has the important role of contributing to whether a woman uses LACMs. Concentrated efforts are needed towards male education and involvement in long acting contraceptive use given that spousal permission was one of the major findings of this study.
